# Optimization of selection for growth in Menz Sheep while minimizing inbreeding depression in fitness traits

**DOI:** 10.1186/1297-9686-45-20

**Published:** 2013-06-19

**Authors:** Solomon Gizaw, Tesfaye Getachew, Aynalem Haile, Barbara Rischkowsky, Johann Sölkner, Markos Tibbo

**Affiliations:** 1 Debre Birhan Agricultural Research Centre, P.O. Box 112, Debre Birhan, Ethiopia; 2 International Centre for Agricultural Research in the Dry Areas, P.O. Box 5466, Aleppo, Syria; 3 BOKU, University of Natural Resources and Life Sciences, 1180, Vienna, Austria; 4 Food and Agriculture Organization of the United Nations, Regional Office for the Near East, P.O. Box 2223, Dokki, Cairo, Egypt

## Abstract

The genetic trends in fitness (inbreeding, fertility and survival) of a closed nucleus flock of Menz sheep under selection during ten years for increased body weight were investigated to evaluate the consequences of selection for body weight on fitness. A mate selection tool was used to optimize in retrospect the actual selection and matings conducted over the project period to assess if the observed genetic gains in body weight could have been achieved with a reduced level of inbreeding. In the actual selection, the genetic trends for yearling weight, fertility of ewes and survival of lambs were 0.81 kg, –0.00026% and 0.016% per generation. The average inbreeding coefficient remained zero for the first few generations and then tended to increase over generations. The genetic gains achieved with the optimized retrospective selection and matings were highly comparable with the observed values, the correlation between the average breeding values of lambs born from the actual and optimized matings over the years being 0.99. However, the level of inbreeding with the optimized mate selections remained zero until late in the years of selection. Our results suggest that an optimal selection strategy that considers both genetic merits and coancestry of mates should be adopted to sustain the Menz sheep breeding program.

## Findings

### Research problem and hypothesis

Selective breeding is expected to reduce fitness of animal populations through its negative effect on genetic diversity, particularly in small closed populations. The reduction in fitness caused by intensive selection for production traits is more severe under random mating systems due to more frequent mating between close relatives [1]. Optimum genetic gains while minimizing inbreeding can be achieved by adopting appropriate selection and mating designs. Such designs include the family mating design [2], where mating permissions are restricted within mating groups, and minimum coancestry mating, which involves individual mate selection that limits the contribution of individuals to future generations [3-5].

A selective breeding program for the Menz sheep breed in Ethiopia has been ongoing in a closed nucleus flock since 1998. Remarkable improvements in body weight have been recorded [6]. The objectives of this paper were to evaluate the current Menz sheep breeding program in terms of genetic trends in fitness (fertility and survival), as related to inbreeding, and to investigate whether the observed genetic gains in body weight could have been achieved with a reduced level of inbreeding by optimizing the actual selection and matings conducted over the project years by adopting the individual mate selection approach.

## Methodology

### Estimation of breeding values

A closed nucleus population of Menz sheep was established in 1998 in the subalpine highlands at the Debre Birhan Agricultural Research Center in Ethiopia. Selection of breeding stocks was based on estimated breeding values (EBV) for yearling weight which were estimated using the best linear unbiased prediction (BLUP) procedure fitting a univariate model. Mating was carried out according to a family mating design, in which random mating of individuals was allowed within mating groups [6,7]. The mating groups were created by dividing the foundation flock into five groups at the beginning of the breeding program. The mating groups remained closed with female replacements coming from within the group, but rams were not permitted to mate in their groups of origin.

Data obtained from the Menz sheep nucleus flock from 1998 to 2008 was used to estimate genetic trends in yearling weight, ewe fertility, pre-weaning lamb survival and level of inbreeding. Ewe fertility and pre-weaning lamb survival are binary traits and are defined respectively, as number of ewes lambing combined as a proxy measure to pregnancy, and as survival of a lamb from birth to weaning at three months. Ewes lambing and lambs surviving were assigned a value of 1, whereas ewes not lambing and lambs not surviving a value of 0. The pedigree data used to estimate breeding values consisted of 2194 animals born from 709 dams and 80 sires, spanning over seven generations of pedigree for yearling weight and survival and six generations for fertility. A total of 1142, 1842 and 2152 records were available for yearling weight, survival and fertility, respectively. Breeding values were estimated for all traits by fitting mixed linear models. This ignores the categorical nature of the binary traits fertility and survival. However, several studies have shown that linear models that assume underlying continuous distributions can be applied to estimate genetic parameters [8,9] and linear and threshold models have given very similar heritability estimates for survival in another Menz sheep population [10]. Breeding values and inbreeding coefficients (*F*) for each animal were estimated simultaneously using WOMBAT [11] by fitting the following univariate model:

$$Y_{i}=X_{i}b_{i}+Z_{i}a_{i}+e_{i}$$

where **Y**_*i*_ is a vector of observations for trait *i*, **b**_*i*_ a vector of fixed effects for trait *i* (sex, season, year and dam age for yearling weight and lamb survival; season, year and age for ewe fertility), **a**_*i*_ a vector of random animal effects for trait *i*, **e**_*i*_ a vector of random residual effects for trait *i* and **X**_*i*_ and **Z**_*i*_ are incidence matrices relating records for trait *i* to fixed and random animal effects, respectively.

### Retrospective optimization

The actual matings carried out in the Menz sheep breeding program were revised in retrospect. For the optimized retrospective selection and mating, we used the actual candidates and parameters in each mating year, with selection on yearling weight and individual mate selection with minimum coancestry using the mate selection tool MateSel version 1.5 [3], based on the principles of optimal contributions of parents to future generations [4,5]. Optimization was attempted by placing a hard constraint on genetic gain and constraining the threshold of progeny inbreeding to 1%. A maximum of five and a minimum of three sires (mating groups) were considered in each mating year. The average EBV and inbreeding coefficients of lambs born in each year of the optimized matings were compared with the actual values obtained in the breeding program.

## Results and discussion

### Observed genetic trends

**Figure 1:**
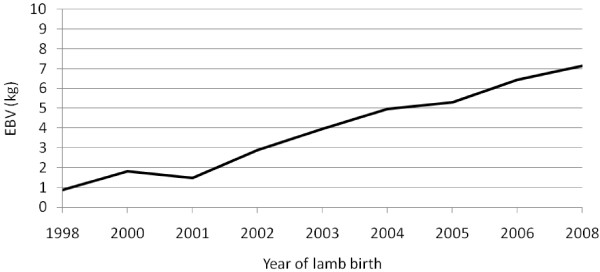
Genetic trends in yearling weight in a closed nucleus population of Menz sheep selected for yearling weight.

**Figure 2:**
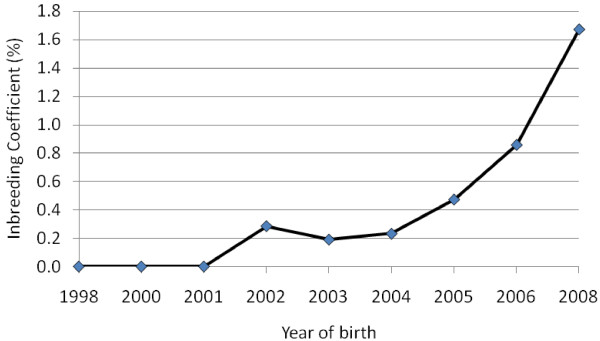
Inbreeding trends in a closed nucleus population of Menz sheep selected for yearling weight.

Average EBV for yearling weight of lambs increased over the years of selection (Figure 1). The average genetic trend per year, calculated by regressing average EBV on year of birth, was 0.81 kg. The average inbreeding coefficient remained zero for the first few generations and below the acceptable level of 1% in later generations (Figure 2). However, the trend increased over generations. The rate of inbreeding (Δ*F*) per generation calculated by regressing *F* on generation number was 0.17%. These results show that a high rate of genetic progress in growth can be achieved with an acceptable increase of less than 1% *F* per generation, at least in the short term.

**Figure 3:**
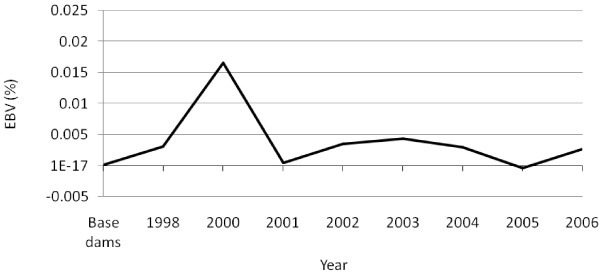
Genetic trends in ewe fertility in a closed nucleus population of Menz sheep selected for yearling weight.

**Figure 4:**
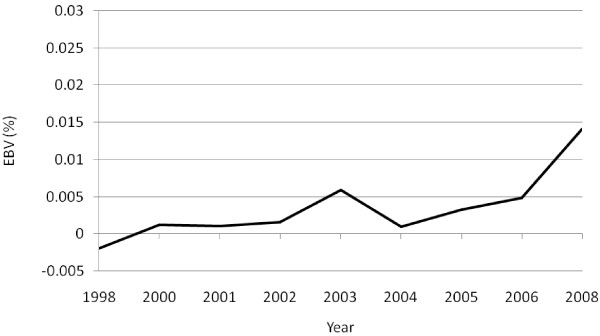
Genetic trends in preweaning survival in a closed nucleus population of Menz sheep selected for yearling weight.

The level of fertility was inconsistent over the period of selection (Figure 3). However, the overall genetic trend was negative, with an average decline in EBV of 0.00026% per generation. Average EBV for survival showed an average increase of 0.016% per generation (Figure 4). Correlations of individual coefficients of inbreeding with EBV for ewe fertility (*r* = −0.023) and lamb survival (*r* = 0.074), taken as indicators of the effect of inbreeding on fitness, were small and statistically not significant (p > 0.05). These findings indicate that genetic progress in growth can be achieved without a significant decline in fitness, at least in the short term, provided that a family mating scheme, rather than a random mating scheme is adopted. Random mating has been experimentally shown to result in a higher level of inbreeding and reduced fitness [1].

### Optimized genetic trends

The genetic gains in yearling weight that were achieved in the optimized retrospective selection and matings were compatible with the observed values (Table 1), the correlation between the average EBV of lambs born from the actual and optimized matings over the years being 0.99. However, the level of inbreeding with optimized mate selections remained zero until late in the years of selection, with only minimal inbreeding in the last two generations (*F* < 1%). This was in contrast with the earlier buildup of inbreeding in the actual selection program. Similarly, the percentage of individuals with inbreeding coefficients greater than 1% reached 25 to 50% in the actual breeding program, compared to only 3 to 5% in the retrospectively optimized mating scheme. Furthermore, among the animals born in 2008, all were inbred in the actual selection program, with *F* ranging from 0.098 to 6.25%, whereas only 45.0% were inbred in the simulated population, with *F* ranging from 0.1 to 1.56%.

**Table 1:** Optimized genetic progress in yearling weight (EBV) and inbreeding (F) compared with values observed in a closed nucleus population of Menz sheep selected for yearling weight

**Birth year**	**Average EBV (kg)**		**Average F (%)**		**Percent progeny with F > 1%**	
	**Optimized**	**Observed**	**Optimized**	**Observed**	**Optimized**	**Observed**
1998	0.31	0.87	0	0	0	0
2000	1.33	1.83	0	0	0	0
2001	1.87	1.49	0	0	0	0
2002	3.36	2.88	0	0.28	0	4.7
2003	4.05	3.97	0	0.19	0	6.0
2004	5.36	4.96	0	0.23	0	6.1
2005	5.94	5.30	0	0.47	0	13.9
2006	6.58	6.44	0.14	0.86	3.1	24.7
2008	7.58	7.16	0.32	1.67	5.1	50.0

## Conclusion

Family mating has proved to be an efficient mating tool to achieve maximum gains in productive traits with minimal losses in fitness traits in the short term. However, a strategy involving optimal mate selection that considers both genetic merits and coancestry of mates should be adopted to sustain the Menz sheep breeding program in the long run.

## Competing interests

The authors declare they have no competing interests.

## Authors’ contributions

SG and TG conducted the breeding program and collected the data. MT suggested the research question for this paper. SG analyzed the data; SG, BR, MT, AH, TG and JS wrote the paper. All authors read and approved the final manuscript.

## References

[B1] Moreno A Salgado C Piqueras P Gutiérrez JP Toro MA Ibanez-Escriche N Nieto B Restricting inbreeding while maintaining selection response for weight gain in *Mus musculus* J Anim Breed Genet 2011 128 276 283 10.1111/j.1439-0388.2011.00933.x 21749474

[B2] Croston D Pollot G Planned sheep production 1994 Oxford: Blackwell Scientific Publications

[B3] Kinghorn BP An algorithm for efficient constrained mate selection Genet Sel Evol 2011 43 4 10.1186/1297-9686-43-4 21251244 PMC3037843

[B4] Meuwissen THE Sonesson AK Maximizing the response of selection with predefined rate of inbreeding: overlapping generations J Anim Sci 1998 76 2575 2583 9814896 10.2527/1998.76102575x

[B5] Meuwissen THE Maximizing the response of selection with a predefined rate of inbreeding J Anim Sci 1997 75 934 940 9110204 10.2527/1997.754934x

[B6] Gizaw S Lemma S Komen H van Arendonk JAM Estimates of genetic trends and genetic parameters for live weight and fleece traits in Menz sheep Small Ruminant Res 2007 70 145 153 10.1016/j.smallrumres.2006.02.007

[B7] Gizaw S Getachew T Tibbo M Haile A Dessie T Congruence between selection of breeding rams based on breeding values for production traits and farmers ram choice criteria Animal 2011 7 995 1001 22440095 10.1017/S1751731111000024

[B8] Phocas F Sapa J Genetic parameters for growth, reproductive performance, calving ease and suckling performance in beef cattle heifers Anim Sci 2004 79 41 48

[B9] Kadarmideen HN Thompson R Coffey MP Kossaibati MA Genetic parameters and evaluations from single and multiple trait analysis of dairy cow fertility and milk production Livest Prod Sci 2003 81 183 195 10.1016/S0301-6226(02)00274-9

[B10] Gizaw S Joshi BK Genetic and non-genetic factors affecting survivability of Menz and Awassi x Menz crossbred sheep in Ethiopia Indian J Anim Sci 2004 74 887 889

[B11] Meyer K WOMBAT - a tool for mixed model analyses in quantitative genetics by REML J Zhejiang Univ Sci B 2007 8 815 821 10.1631/jzus.2007.B0815 17973343 PMC2064953

